# FPGA Implementation of Optimal 3D-Integer DCT Structure for Video Compression

**DOI:** 10.1155/2015/204378

**Published:** 2015-10-27

**Authors:** J. Augustin Jacob, N. Senthil Kumar

**Affiliations:** ^1^Kamaraj College of Engineering and Technology, Virudhunagar 626001, India; ^2^Mepco Schlenk Engineering College, Sivakasi 626001, India

## Abstract

A novel optimal structure for implementing 3D-integer discrete cosine transform (DCT) is presented by analyzing various integer approximation methods. The integer set with reduced mean squared error (MSE) and high coding efficiency are considered for implementation in FPGA. The proposed method proves that the least resources are utilized for the integer set that has shorter bit values. Optimal 3D-integer DCT structure is determined by analyzing the MSE, power dissipation, coding efficiency, and hardware complexity of different integer sets. The experimental results reveal that direct method of computing the 3D-integer DCT using the integer set [10, 9, 6, 2, 3, 1, 1] performs better when compared to other integer sets in terms of resource utilization and power dissipation.

## 1. Introduction

Nowadays most video compression algorithms rely on reducing the spatial and temporal redundancy by motion compensation and prediction. However these algorithms are complex and no symmetry exists between encoding and decoding block. This has made implementation of the algorithm more complex. 3D-DCT based video coding [1] is considered as an alternate to the existing standard video compression algorithms. It eliminates some of the problems like blocking effect caused by motion estimation algorithm, which is lossy and time-consuming [2]. For a video sequence that involves fast motion object, motion estimation may not yield correct motion vector since full search cannot be done in a given video stream.

Few research efforts are made to enhance the 3D-DCT based video codec [3–5] and made comparable to the standard video compression algorithm. If implementable structure exists for 3D-integer DCT, that will further accelerate the encoding process. A lossy compression scheme has been developed by Zaharia et al. [6] that apply 3D-DCT for compressing 3D integral images and they showed that it outperforms the JPEG standard. Even though recent compression standards developed using discrete wavelet transform outperform the JPEG standard, DCT is the preferred one, because fast computation structures exist for DCT. It reflects the need for proposing new hardware for 3D-integer DCT. However no attempt has been made to implement 3D-integer DCT algorithm. It is essential to find the suitability of 3D-DCT based video coders in real time application by analyzing the hardware complexity.

Most standard video compression algorithms like MPEG and H.26X adopt DCT as part of their standard. This had led to the development of many fast 1D- and 2D-DCT algorithms. The fundamental aim behind the development of new algorithm for DCT is to reduce the number of multiplications and additions. In order to compute DCT for a given input sequence of length *N* it requires *N*2 multiplications and *N*  (*N* − 1) additions. The fast DCT algorithm stated in [7] reduces the computational complexity to (*N*/2) log_2_^*N*^ multiplications and *N* log_2_ ⁡*N* additions. A few algorithms and implementation structure exist for computing real valued 1D-DCT and 2D-DCT [8–23]. Among them the algorithm presented by Prado and Duhamel [16] is given significant importance because the study reveals that if an optimal algorithm is obtained for 1D-DCT then the extension to the corresponding 2D-DCT and 3D-DCT algorithm will also be optimal. However implementing the real value transform becomes more complex since the need of floating point multiplier is unavoidable even if it consumes more resources. Cham et al. [24] have presented a simplified algorithm that first converts the floating point to fixed point and then performs DCT. However exact energy transformation will not happen in this case because of the floating to fixed point conversion. The errors occurring during the computation of 1D-DCT are propagated to the third dimension.

Currently DCT with integer coefficients are of great interest, because the design is simpler and implemented more efficiently. An improvement over traditional real and fixed point implementation was proposed by Edirisuriya et al. [25]. In this paper DCT was computed using integer values. So there is no need to design floating point multiplier that consumes more resource and time. The survey undoubtedly shows the usage of integer DCT in 3D-DCT based video and image compression algorithms. However efforts to design the hardware for 3D-integer DCT are rare in the literature. A few approximation methods are available for deriving the equivalent integer DCT from real value DCT. It is classified as indirect or C-matrix transform method proposed by Kwak et al. [26] and direct method by Pei and Ding [27]. In these papers the two approximation methods (direct and indirect) are considered for analysis and optimal integer set for computing 3D-integer DCT is determined based on MSE and coding efficiency.

Finally based on power dissipation and resource utilization optimal structure for 3D-integer DCT is determined.

## 2. ****3D-Discrete Cosine Transforms

The discrete cosine transform (DCT) is a member of a family of sinusoidal unitary transforms. It found applications in digital signal processing and particularly in image/video compression. The family of discrete trigonometric transforms consists of 8 versions of DCT. Each transform is identified as even or odd and of types I, II, III, and IV. All present image and video processing applications involve only even types of the DCT. In particular DCT-II received much attention in video compression applications because of its high energy packing ability and there exist fast computation structures to compute DCT-II. So throughout the text DCT-II was mentioned as DCT. Equation (1) defines the one-dimensional-DCT and inverse DCT for a finite duration signal *f* of length *N*_*r*_ as

(1) FR=2Nrfr∑r=0Nrfrcos⁡2r+1Rπ2Nr0≤r≤Nr−1fr=2Nrfr∑R=0NrFRcos⁡2r+1Rπ2Nr0≤R≤Nr−1,

where

(2) $$\begin{array}{c} f\left[\;r\;\right]=\left\{\begin{array}{cc} \frac{1}{\sqrt{2}}, & r=0\\ 1, & r=1,2,\ldots ,N_{r}-1. \end{array}\right. \end{array}$$

Usually image and video frames are two-dimensional in nature. Because of the orthogonality and separability property, DCT can be extended to two dimensional forms. The 2D-DCT for a block of pixels of size *N* × *N* whose intensity values range between 0 and 255 is defined in

(3) FR,C=4Nr·Ncfrfc∑r=0Nr ∑c=0Ncfr,ccos⁡2r+1Rπ2Nr·cos⁡2c+1Cπ2Ncfr,c=4Nr·Ncfrfc∑R=0Nr ∑C=0NcFR,C·cos⁡2r+1Rπ2Nrcos⁡2c+1Cπ2Nc,

where *r*, *c*, *R*, *C* = 0,1,…, *N*_*r*_/*N*_*c*_ − 1. Consider

(4) $$\begin{array}{c} f\left[\;r,c\;\right]=\left\{\begin{array}{cc} \frac{1}{\sqrt{2}}, & \text{for }r,c=0\\ 1, & \text{otherwise.} \end{array}\right. \end{array}$$

The equation for computing 2D-DCT is extended along the temporal domain to get the required expression for computing 3D-DCT. It is defined in (5) and (7). Consider

(5) FR,C,D=8Nr·Nc·Ndfrfcfd∑r=0Nr ∑c=0Nc ∑d=0Ndfr,c,d·cos⁡2r+1Rπ2Nrcos⁡2c+1Cπ2Nc·cos⁡2d+1Dπ2Nd,

where

(6) $$\begin{array}{c} f_{r},f_{c},f_{d}=\left\{\begin{array}{cc} \frac{1}{\sqrt{2}}, & \text{for }r,c,d=0\\ 1, & \text{otherwise}, \end{array}\right. \end{array}$$

where *F*(*R*, *C*, *D*) and *f*(*r*, *c*, *d*) represent the frequency domain and time domain intensity values, respectively. Correspondingly the expression for finding inverse 3D-DCT is given as shown below:

(7) fr,c,d=8Nr·Nc·Ndfrfcfd∑R=0Nr ∑C=0Nc ∑D=0NdFR,C,D·cos⁡2r+1Rπ2Nrcos⁡2c+1Cπ2Nccos⁡2d+1Dπ2Nd.

## 3. Integer Approximation of 3D-DCT Using Indirect Method

In indirect method integer values are obtained using other orthogonal transforms like the Walsh-Hadamard transform. DCT can be implemented using WHT through a conversion matrix shown in

(8) C^=TN·W^NTTN=C^N·W^NT,

where $\hat{C}$ represents discrete cosine transform and *T*_*N*_ is the conversion matrix which converts the Walsh domain vector (${\hat{W}}^{T}$) into DCT domain. In indirect method there are totally 11 different elements in the conversion matrix. Substitution of variable for each nonzero element in the matrix results in 11 variables denoted as {*A*, *B*, *C*, *D*, *E*, *F*, *G*, *H*, *I*, *J*, *K*}. It is represented in (9), where ${\hat{T}}_{8}$ is approximated conversion matrix:

(9) $$\begin{array}{c} {\hat{T}}_{8}=\frac{1}{A}\left(\begin{array}{cccccccc} A & 0 & 0 & 0 & 0 & 0 & 0 & 0\\ 0 & A & 0 & 0 & 0 & 0 & 0 & 0\\ 0 & 0 & B & C & 0 & 0 & 0 & 0\\ 0 & 0 & -C & B & 0 & 0 & 0 & 0\\ 0 & 0 & 0 & 0 & D & -E & H & I\\ 0 & 0 & 0 & 0 & F & G & -J & K\\ 0 & 0 & 0 & 0 & -K & J & G & F\\ 0 & 0 & 0 & 0 & -I & -H & -E & D \end{array}\right). \end{array}$$

Preserving the signs of the element of ${\hat{T}}_{8}$ a search was made to find suitable integer values. Also it has to satisfy the following algebraic equations:

(10) DF−EG−JH+IK0

(11) DK+EJ−HG−IF0

(12) B2+C2D2+E2+H2+I2=F2+G2+J2+K2=A2.

Equations (10) and (11) are conditions of orthogonality and they ensure that rows of ${\hat{T}}_{8}$ are orthogonal to each other. Equation (12) is for normality condition. In order to make ${\hat{T}}_{8}$ resemble those of real valued transform constraints are set on the variables *A*, *B*, *C*, *D*, *E*, *F*, *G*, *H*, *I*, *J*, *K*. The magnitudes of the elements in *T*_8_ are compared and the following inequalities are obtained:

(13) D>G>J>H>K>F>I>E>0

(14) J>C≥H−1

(15) B≥D−1

(16) A>B.

**Figure 1:**
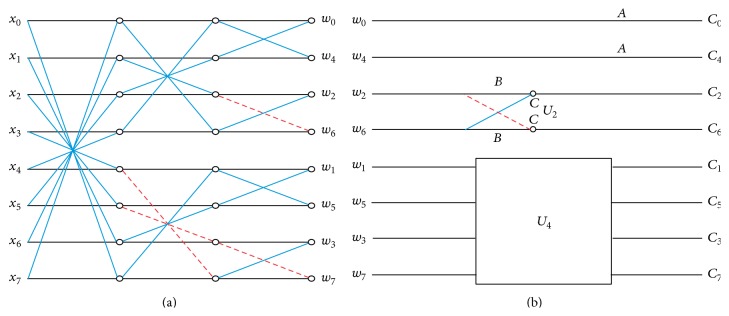
((a), (b)) The signal flow graph of fast integer DCT using indirect method.

All the integer solutions satisfying (10) to (12) under constraints given by (13) to (16) will guarantee that the approximated conversion matrix ${\hat{T}}_{8}$ is orthonormal and close to the original conversion matrix *T*_8_. The generalized signal flow graph of integer approximation using indirect method is given in Figure 1, where

(17) U2=BC−CB,U4=D−EHIFG−JK−KJGF−I−H−ED.

In Figure 1 the lines indicated in blue color represent addition and dotted lines indicated in red color represent subtraction. Additional information regarding integer approximation can be found in the work done by Britanak et al. [28].

## 4. Integer Approximation Using Direct Method

In direct method equivalent integer values are obtained directly and it replaces the rational number in the DCT matrix. The approximated integer cosine transform matrix is given by

(18) $$\begin{array}{c} C_{8}^{\text{IDCT}}=Q_{8}\cdot V_{8}, \end{array}$$

where *Q*_8_ is a diagonal matrix with normalization factors on its main diagonal and *V*_8_ is an integer matrix. It is seen that totally there are 7 different elements in the DCT matrix. The same variables are used to represent the elements in the conversion matrix having the same magnitude. Substituting a variable for each nonzero element in the matrix results in 7 variables denoted as {*A*, *B*, *C*, *D*, *E*, *F*, *G*} as it is shown in (19). Set of inequalities are formed so that orthogonality and normality property of DCT matrix is preserved in the integer domain. Consider

(19) C8IDCT=Q8GGGGGGGGABCD−D−C−B−AEF−F−E−E−FFEB−D−A−CCAD−BG−G−GGG−G−GGC−ADB−B−DA−CF−EE−F−FE−EFD−CB−AA−BC−D

(20) AB−C−DB+C=0

(21) A>B>C>D>0

(22) A>E>F>0

(23) A>G>0.

**Figure 2:**
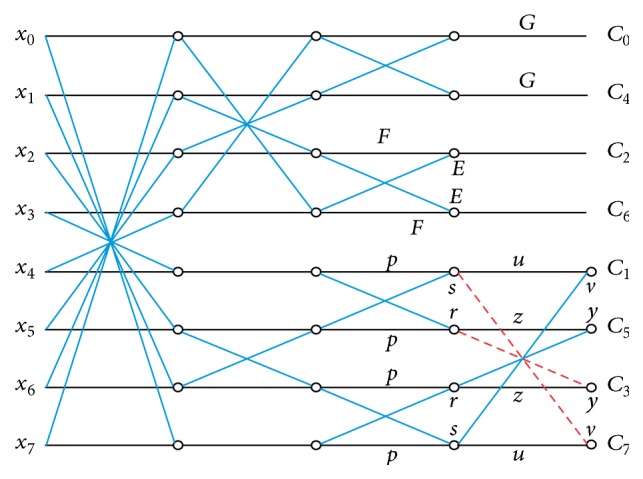
The signal flow graph of fast integer DCT using direct method.

By solving (20) under set of constraints described in (21) to (23), different integer solutions set are obtained [22]. Integer sets with low mean squared error (MSE) and high transform coding efficiency (*n*) are preferred to get the optimal solution for 3D-integer DCT. Fast computation structures are obtained by recursive sparse matrix factorization method. The generalized signal flow graph of integer approximation using direct integer DCT is given in Figure 2, where the parameters *p*, *r*, *s*, *u*, *v*, *y*, *z* are integers or dyadic rational.

## 5. Criteria for Evaluation of Approximated Integer DCT

In order to evaluate the approximation error between the integer DCT and original transform matrix and to measure the difference in performance in data compression, some theoretical criteria are needed. For this purpose, the input signal is frequently modeled as a first-order stationary Markov process (Markov-1) with zero-mean, unit variance, and adjacent interelement correlation coefficient *ρ* chosen between zero and one. Then, the input signal **X** is defined by a covariance matrix *R*_*x*_, whose elements are given by

(24) $$\begin{array}{c} {\left[\;R_{x}\;\right]}_{ij}=\rho^{\left|i-j\right|}. \end{array}$$

The matrix *R*_*x*_ is symmetric and Toeplitz. The covariance matrix *R*_*y*_ of the transformed vector **y**, where **y** = *A* **x**, is obtained from (25):

(25) $$\begin{array}{c} R_{y}=AR_{x}A^{T}. \end{array}$$

## 6. Mean Squared Error

For the evaluation of approximation error between the approximated and original transform matrix, the parameter mean squared error (MSE) was used. It is defined as follows. Let us assume that *U*_*N*_ is the original transform matrix and ${\hat{U}}_{N}$ is its approximation. Then, for a given input vector **X** of length *N*, the error vector is

(26) $$\begin{array}{c} e=U_{N}x-{\hat{U}}_{N}x=\left(\;U_{N}-{\hat{U}}_{N}\;\right)x=Dx. \end{array}$$

From (26), the MSE between the original and approximated transform can be defined by

(27) ϵ1NEeeT1NExTDTDx=1NETraceDxxTDT=1NTraceDRxDT,

where *R*_*x*_ is the covariance matrix of the input signal **X**. Thus, to maintain the compatibility between the original and approximated transform, the MSE should be minimized.

## 7. Transform Efficiency

Equation (28) defines the transform efficiency:

(28) $$\begin{array}{c} \eta =\frac{\sum_{i=0}^{N-1}\left|r_{ii}\right|}{\sum_{i=0}^{N-1}\sum_{j=0}^{N-1}\left|r_{ij}\right|}100, \end{array}$$

where *r*_*ij*_ are elements of *R*_*y*_. The transform efficiency measures the decorrelation ability of the transform. The optimal KLT converts signal into completely uncorrelated coefficients and it has transform efficiency *η* = 100 for all values of *ρ*, while the DCT has transform efficiency *η* = 93.9911 for the correlation coefficient *ρ* = 0.95.

## 8. Structure for Computing 3D-Integer Discrete Cosine Transform

**Figure 3:**
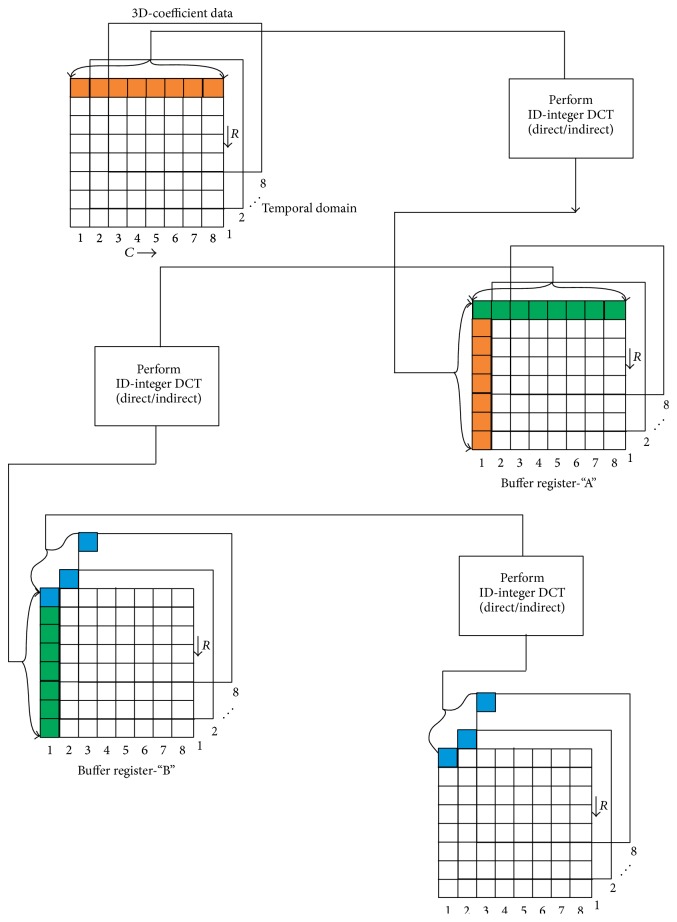
Block diagram of computing 3D-integer DCT.

In order to reduce the hardware complexity optimal integer sets from direct and indirect integer approximation are chosen based on number of multiplications/additions. The structure that possesses minimum complexity is considered for computing 3D-integer DCT by taking 1D-integer DCT along row, column, and temporal domain. The block diagram of the proposed 3D-integer DCT is shown in Figure 3. To compute 3D-integer DCT for the cube of dimension, say, 8 × 8 × 8, the 1D-integer DCT is initially performed along the row wise and the computed values are stored in buffer “*A*” along the column wise. The process is repeated for all the rows of the cube starting from frame 1 to frame 8. To have clear visualization rows are marked with the same color, as shown in Figure 3. Here the buffer size and cube size are identical. The structure for computing DCT may be from either direct method or indirect method. Similarly 1D-integer DCT is computed for the values stored in buffer “*A*” along the row wise and the results are stored along the column wise in buffer “*B*,” this result in 2D-integer DCT. Then perform one more 1D-integer DCT for the values stored in buffer “*B*” along the temporal direction that gives the 3D-integer DCT value as shown in Figure 3.

## 9. Experimental Results

### 9.1. Determination of Optimal Integer Set for Computing 3D-Integer Discrete Cosine Transform

In order to determine the optimal integer set the performances of the proposed 3D-IDCT are compared against the existing real valued transforms with respect to MSE and transform coding efficiency. Different possible integer solutions exist for both the direct and indirect method of computing 1D-IDCT and it is subjected to computing 3D-IDCT. The MSE and transform coding efficiency of the corresponding integer sets along with the computational complexity are listed in Tables 1 and 2.

The integer solutions whose MSE and coding efficiency are very close to real value transform are considered for FPGA implementation. Also it is observed that though the integer set with higher bit solutions (5, 6, 7, and 8) yield low MSE and high coding efficiency, it is not preferred for implementation. Because when computing 3D-integer DCT the size of registers (buffers “*A*” and “*B*”) holding intermediate values becomes larger for higher bit solutions that directly increases the computational complexity (ie) higher bit length multiplier is required. Further, it is noted that for integer set having zero and one, as one of the elements, variation in multiplication/additions is observed.

Here the number of multiplications and additions is estimated based on the structure shown in Figure 3. With reference to the results obtained in Tables 1 and 2 the optimal integer set is determined to be [10, 9, 6, 2, 3, 1, 1] because this integer set yields relatively low MSE and high coding efficiency when compared to real value transform.

Further it was observed that if optimal integer set is used to encode the video sequence instead of real value 3D-DCT there is no much deviation in PSNR value. However it is noticed that the deviation is proportional to the MSE of the corresponding integer set. For the optimal integer set the maximum degradation in PSNR value was found to be 0.01 db.

## 10. FPGA Implementation of 3D-IDCT

The hardware design for computing 3D-integer DCT for a block of data 8 × 8 × 8 using the integer set [10, 9, 6, 2, 3, 1 and 1] was coded in Verilog Hardware Description Language. The functional behavior of the design was tested in Xilinx ISE simulator with sample data set. Simulations are also performed using MATLAB for the same data set for correctness. The design was mapped on to Artix-7 FPGA board. The Artix-7 belongs to 28-nanometer (nm) process technology designed for low power products used in portable communication devices. The maximum DC value of 3D-DCT was found to be 4000. If normalization factors are neglected, in integer domain maximum of 17 bits are required to hold the 3D-integer DCT value.

**Figure 4:**
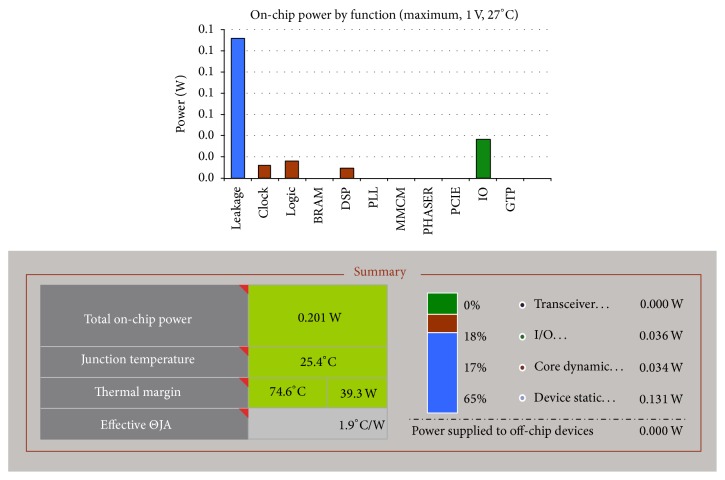
On-chip power utilization of 3D-integer DCT for the integer set [10, 9, 6, 2, 3, 1, 1].

As the value of elements in the integer set increases, then bit length of the processing elements also increases to show that the least resources are utilized for the integer set that has shorter bit values. Synthesis was performed for the integer set [13, 12, 5, 12, 0, 0, 12, 4, 3, 3, 4] and comparison has been made with the optimal integer set. From the device utilization summary shown in Table 3 it was noticed that higher resources are utilized for the integer set [13, 12, 5, 12, 0, 0, 12, 4, 3, 3, 4]. It is due to the fact that, for computing 3D-integer set, this integer set requires 25 bits; however for optimal integer set it requires only 17 bits. So when bit length of the integer set increases then bit length of computational unit (multiplication/addition) also increases that leads to higher resource utilization. In order to estimate the power consumption of the design Xilinx Power Estimator (XPE) tool was used. The distribution of on-chip power and total power of the design is shown in Figure 4.

The total on-chip power reflects the heat dissipated from the chip. If the device operates at 100 MHz clock, with the total on-chip power of 0.201 W, then the junction temperature is 25.4°C and it is well below the thermal margin of the target FPGA device. Also a comparison has been made between the existing fixed point 2D-DCT algorithm based on Loffler method [22] and the proposed 3D-integer DCT algorithm in terms of device utilization. It is identified that twelve instances of fixed point 2D-DCT Loffler structures are needed to compute a fixed point 3D-DCT algorithm in accordance with the fact that resource utilization is calculated and it is given in Table 4.

It is clearly seen from Table 4 that the proposed 3D-integer DCT algorithm with optimal integer set [10, 9, 6, 2, 3, 1, 1] outperforms the fixed point 3D-DCT algorithm based on Loffler method [22].

## 11. Conclusion

In this paper various integer sets from different approximation methods for converting real to integer value transforms are analyzed in terms of MSE and coding efficiency. Based on that, optimal integer set is chosen for computing 3D-integer DCT. Further if optimal integer set was adopted to encode the video sequence, then the deviation in PSNR with respect to real value DCT was found to be 0.01 db. Also a new hardware structure for computing the 3D-integer DCT is proposed and implemented the same in FPGA board. The synthesis results reveal that the least resources are utilized for the integer set that has shorter bit values. Also based on number of additions and multiplications variation in resource utilization is observed. The experimental results reveal that direct method of computing the 3D-integer DCT using the integer set [10, 9, 6, 2, 3, 1, 1] performs better when compared to other integer sets in terms of resource utilization and power dissipation.

## Figures and Tables

**Table 1:** MSE and coding efficiency of 3D-integer DCT computed via WHT.

3D-integer DCT coefficients (*A*, *B*, *C*, *D*, *E*, *F*, *G*, *H*, *I*, *J*, *K*)	Mean squared error	Coding efficiency (*η*)
Real valued transform	0	74.8830

13, 12, 5, 12, 0, 0, 12, 4, 3, 3, 4 Multiplications: 54 Additions: 102	0.0236	72.2229

34, 30, 16, 31, 1, 7, 25, 13, 5, 19, 11 Multiplications: 60 Additions: 114	0.0031	73.0016

39, 36, 15, 35, 2, 8, 30, 16, 6, 19, 14 Multiplications: 66 Additions: 114	0.0011	74.1108

**Table 2:** MSE and coding efficiency of 3D-integer DCT computed using direct method.

S. number	3D-integer DCT coefficients (*A* *B* *C* *D* *E* *F* *G*)	Mean squared error	Coding efficiency (*η*)
	Real valued transform	0	74.8830

*3-bit solution* (multiplications: 30, additions: 78)			
1	5 3 2 1 3 1 1	0.0133	70.1868
2	7 4 3 1 3 1 1	0.0155	69.1980

*4-bit solution* (multiplications: 48, additions: 78)			
3	10 9 6 2 3 1 1	0.0014	74.8115
4	14 12 9 2 3 1 1	0.0024	74.0339
5	12 10 6 3 3 1 1	0.0022	73.3292
6	15 12 8 3 3 1 1	0.0014	73.9534

*5-bit solution* (multiplications: 48, additions: 78)			
7	24 21 15 4 3 1 1	0.0017	74.4362
8	25 21 14 5 3 1 1	0.0010	74.4961
9	25 24 16 5 3 1 1	0.0026	74.7795
10	26 24 16 6 3 1 1	0.0019	74.4872

*6-bit solution* (multiplications: 48, additions: 78)			
11	45 39 26 9 3 1 1	0.0011	74.7986
12	45 42 28 9 3 1 1	0.0019	74.9107
13	55 51 34 11 3 1 1	0.0018	74.9117
14	55 48 32 11 3 1 1	0.0011	74.8522

*7-bit solution* (multiplications: 48, additions: 78)			
15	65 57 38 13 3 1 1	0.0011	74.8726
16	75 66 44 15 3 1 1	0.0011	74.8847
17	85 75 50 17 3 1 1	0.0012	74.8940
18	120 105 70 24 3 1 1	0.0011	74.8650

*8-bit solution* (multiplications: 48, additions: 78)			
19	175 153 102 35 3 1 1	0.0011	74.8611
20	185 162 108 37 3 1 1	0.0011	74.8677
21	230 201 134 46 3 1 1	0.0011	74.8589
22	250 219 146 50 3 1 1	0.0011	74.8690

**Table 3:** Device utilization summary.

Device utilization	Optimal integer set [10, 9, 6, 2, 3, 1, 1]	Sample integer set [13, 12, 5, 12, 0, 0, 12, 4, 3, 3, 4]
Number of slice registers (out of 437200)	975	1507

Number of slice LUT (out of 218600)	5396	7054

Number of fully used LUT-FF pairs (out of 6014)	357	146

Number of bonded IOBs (out of 250)	213	269

Number of DSP slices (out of 900)	22	120

Clock	100 MHz	104.46 MHz

Computational complexity Multiplications/additions	48/78	54/102

**Table 4:** Comparison of device utilization summaries of the proposed method.

Device utilization	Fixed point 3D-DCT algorithm based on Loffler method [22]	Proposed 3D-integer DCT algorithm with the optimal integer set [10, 9, 6, 2, 3, 1, 1]
Number of slice registers	5112	975
Number of slice LUT	27360	5396
Number of fully used LUT-FF pairs	15708	357
Bonded IOBs	274	213
